# Associations between Well-Being State and Match External and Internal Load in Amateur Referees

**DOI:** 10.3390/ijerph18063322

**Published:** 2021-03-23

**Authors:** Eñaut Ozaeta, Javier Yanci, Carlo Castagna, Estibaliz Romaratezabala, Daniel Castillo

**Affiliations:** 1 Faculty of Education and Sport, University of the Basque Country UPV/EHU, 01007 Vitoria-Gasteiz, Spain; eozaeta001@gmail.com; 2 Society, Sports and Physical Exercise Research Group (GIKAFIT), Physical Education and Sport Department, Faculty of Physical Activity and Sports Science, University of the Basque Country UPV/EHU, 01007 Vitoria-Gasteiz, Spain; estibaliz.romaratezabala@ehu.eus; 3 Football Training and Biomechanics Laboratory, Technical Department, Italian Football Federation (FIGC), 50135 Florence, Italy; castagnac@libero.it; 4 Faculty of Health Sciences, University Isabel I, 09003 Burgos, Spain; daniel.castillo@ui1.es

**Keywords:** field referees, assistant referees, psychometric variables, heart rate, physical demands

## Abstract

The main aim of this paper was to examine the association between prematch well-being status with match internal and external load in field (FR) and assistant (AR) soccer referees. Twenty-three FR and 46 AR participated in this study. The well-being state was assessed using the Hooper Scale and the match external and internal loads were monitored with Stryd Power Meter and heart monitors. While no significant differences were found in Hooper indices between match officials, FR registered higher external loads (*p* < 0.01; ES: 0.75 to 5.78), spent more time in zone 4 and zone 5, and recorded a greater training impulse (TRIMP) value (*p* < 0.01; ES: 1.35 to 1.62) than AR. Generally, no associations were found between the well-being variables and external loads for FR and AR. Additionally, no associations were found between the Hooper indices and internal loads for FR and AR. However, several relationships with different magnitudes were found between internal and external match loads, for FR, between power and speed with time spent in zone 2 (*p* < 0.05; r = −0.43), ground contact time with zone 2 and zone 3 (*p* < 0.05; r = 0.50 to 0.60) and power, speed, cadence and ground contact time correlated with time spent in zone 5 and TRIMP (*p* < 0.05 to 0.01; r = 0.42 to 0.64). Additionally, for AR, a relationship between speed and time in zone 1 was found (*p* < 0.05; r = −0.30; CL = 0.22). These results suggest that initial well-being state is not related to match officials’ performances during match play. In addition, the Stryd Power Meter can be a useful device to calculate the external load on soccer match officials.

## 1. Introduction

Soccer is an intermittent team sport which is officiated by field referees (FR) in cooperation with assistant referees (AR) during official matches. Since FR and AR adopt distinguished roles in order to control the players’ behaviors, their physical demands are different characterized by linear and multidirectional movement, respectively [1]. Previous studies have shown that FR and AR are exposed to different external and internal loads during match play. As such, FR covered greater distance (≈10 km) than AR (≈6 km) during Union of European Football Associations (UEFA) Champions League and U-21 international matches [2,3]. Additionally, FR covered more distance at high intensity velocity (>13 km·h^−1^) (2872.8 ± 422.4 m) in comparison to AR (771.4 ± 170.8 m) [4]. Likewise, FR recorded a percentage of 85.6% of their maximum heart rate (HRmax) whereas AR showed a 75.3% of their HRmax [3]. To date, the external loads have been collected by means of global positioning systems (GPS) or by multicamera video tracking, which are expensive and, consequently, not easy to use in amateur refereeing [5,6]. For this reason, there is a need to know whether other, cheaper devices are able to measure external loads. Fortunately, the Stryd Power Meter has been demonstrated as a valid and reliable device to measure external load in other sports modalities [7,8], so it could be interesting to analyze if Stryd is sensitive enough to record the differences between FR and AR during competitions. In addition, Stryd allows for the collection of other external variables, such as vertical oscillation and stiffness, which it is not possible to record with the GPS. This additional information could be relevant in order to understand the external loads encountered by FR and AR during match play.

Although it has been observed that the variables of internal and external loads are associated, most of the studies indicate that these associations are low or moderate [9,10]. During amateur soccer matches, moderate correlations were found between Edwards’ heart-rate-derived training impulse (TRIMP) and the total distance covered by both FR (r = 0.35; ± 0.41) and AR (r = 0.32; ± 0.28) [9]. In addition, for professional FR, no significant associations were found between TRIMP and the total distance covered (r = 0.22, *p* > 0.05) [10], nor they were observed between the mean heart rate (HR_mean_) within a 5 min time period and high intensity activities (>13 km·h^−1^) in the same period time for AR [11]. Given the reported moderate association, valid and reliable metrics depicting internal and external load variables are necessary to understand soccer officials’ match demands. Likewise, considering that previous investigations quantifying the match loads have been carried out with GPS and video tracking systems, it would be interesting to know if the variables obtained with Stryd correlate with internal match loads on amateur FR and AR.

One of the main goals of match officials is to achieve optimal physical condition for officiating matches in order to ensure their successful participation. Unfortunately, to our knowledge, no scientific studies have investigated the weekly training loads supported by match officials as well as their state of preparation before match play. In soccer the well-being state has been recorded by the Hooper scale among other methods [12,13]. The Hooper index and its subsequent subsets have been used to describe players’ wellness as well-being perceptions of muscle soreness, fatigue, stress and sleep quality in order to assess how soccer players cope during official matches [14,15]. No studies have assessed the well-being of match officials in soccer. Therefore, it is important to determine if well-being is associated with match variables that could affect their external and internal match loads. In the same way, it is necessary to learn if the initial state of the FR and AR could influence their external and internal match loads. Previous investigations carried out in team sports have observed that the initial state, measured with the Hooper scale, was associated with the warm-up load and match activities, showing that higher stress and bad sleep levels were associated with a worse performance on a 5–15 m change of direction ability testing in handball players. Additionally, higher stress and muscle soreness levels were associated with higher perceived exertion [16]. Considering the influence of the well-being state on players’ physical performance, it is necessary to investigate the relationship of the initial state and match load in match officials.

Considering the relevance of monitoring external loads by new devices and the necessity to assess the well-being state of match officials before the competition, the aim of this study was: (1) to compare the well-being status (i.e., Hooper indices) of FR and AR before match play, and their external and internal loads during official matches; (2) to analyze whether the initial well-being status is associated with the external and internal match loads for FR and AR; and (3) to analyze the relationship between internal and external match load for FR and AR. 

## 2. Materials and Methods

### 2.1. Participants

Sixty-nine male match officials who officiated soccer matches in *División de Honor* (Vizcaya, Spain) during the 2019–2020 competitive season, participated in our study, of whom 23 were FR (age: 25.65 ± 3.30 year; height: 173.4 ± 3.8 cm; body mass: 64.86 ± 5.82 kg; body mass index, BMI: 21.56 ± 1.67 kg·m^−2^) and 46 were AR (age: 23.11 ± 4.15 year; height: 178.1 ± 4.3 cm; body mass: 72.84 ± 6.67 kg; BMI: 22.94 ± 1.87 kg·m^−2^). Match officials had at least three years of officiating experience at this competitive-level. All participants trained at least two times a week and were involved in refereeing on average twice per month. Subjects were informed of the benefits and risks of the investigation prior to signing an institutionally approved informed consent document to participate in the study. This investigation was performed in accordance to the Declaration of Helsinki and was approved by the Ethics Committee of The University of the Basque Country (Code: M10/2018/289).

### 2.2. Procedures

A descriptive, comparative and correlational design was used to examine the differences and the relationships between well-being variables, external and internal match loads. Prematch, FR and AR separately pointed a number from 1 to 7 for each Hooper scale variable (i.e., sleep, fatigue, stress and muscle soreness) [17] and following these measures officials performed a 10 min warm-up consisting of running, stretching, short sprints and progressive sprints. Throughout the each of the considered matches, the external (distance, power, speed, cadence, vertical oscillation, ground contact time and stiffness) and internal match load (time spent on 5 arbitrary HR zones and TRIMP) were recorded. Data was collected during 23 official in season matches (i.e., from December to February). All matches were played between 11am and 5 pm.

The Hooper questionnaire was completed before the warm-up in order to assess how the participants felt before the match. Every match official declared their feelings individually and pointed to a number from 1 (very, very good) to 7 (very, very bad) for each Hooper questionnaire item [17]. The Hooper index was calculated as the sums of scores of the four Hooper questionnaire items.

### 2.3. Measures

External match-loads: officials’ external loads were monitored using a Stryd Power Meter (Stryd, Inc., Boulder, CO, USA) placed over the right soccer boot with a plastic clip regardless their lower limb dominance [8]. Stryd Power Meter was used to carry out this study because the device’s capacity for repeatability has been demonstrated (systematic error of measurement, SEM ≤ 12.5 W, CV ≥ 4.3%, ICC ≤ 0.989) [7]. The device was activated in accordance with the manufacturer’s recommendations aiming to record data offline. The 15 min half-time intervals were excluded from the statistical analysis. The external match load data were collected as measures of total distance covered (km), average power (W), average speed (km·h^−1^), average cadence (steps per min), average vertical oscillation (cm), average ground contact time (m·s^−1^) and average stiffness (KN·m^−1^).

Internal match-loads: match official’s HR was recorded during the whole match with a Polar Team 2 device (Polar Team System™, Kempele, Finland) at 1 s intervals. The 15 min half-time intervals were excluded from the statistical analysis. The TRIMP based on Edwards (1993) was calculated by the sum of the values obtained multiplying the time spent in 5 arbitrary HR zones (from zone 1 to zone 5) by the number of each zone [18]. The zones were calculated as a percentage of the peak HR (HR_peak_) obtained during the whole match [19]. TRIMP is represented in arbitrary units (AU).

### 2.4. Statistical Analyses

Results are presented as means ± standard deviations. Normal distribution and homogeneity of variances was tested using the Kolmogorov–Smirnov and Levene tests. Parametric tests were performed when data was normally distributed (vertical oscillation, stiffness, zone 3 and TRIMP), whereas equivalent nonparametric tests were used when data violated the assumption of normality (sleep, fatigue, stress, muscle soreness, total distance, power, speed, cadence, ground contact time, zone 1, zone 2, zone 4 and zone 5). The intermatch officials’ coefficient of variation (CV) was used to assess the variability of the Hooper indices and external and internal match loads by the formula CV = (SD·mean-1) × 100 [20]. Student’s t-test for independent samples or U Mann–Whitney test was performed in order to evaluate mean differences between FR and AR in Hooper indices, external and internal match loads. Practical significance was assessed by Cohen’s effect size (ES) [21]. ES of above 0.8, between 0.8 and 0.5, between 0.5 and 0.2, and lower than 0.2 were considered large, moderate, small, and trivial, respectively. Product moment correlation coefficient with a 90% confidence interval (CI) was used to examine the relationship between external match loads and Hooper indices and internal match loads. When at least one nonparametric variable was analyzed Spearman (Rho) was used, and when all of the analyzed variables were parametric variables Pearson (r) was used. The magnitude of the correlation was determined as trivial: r < 0.1, low; 0.1–0.3, moderate; 0.3–0.5, large; 0.5–0.7, very large; 0.7–0.9, nearly perfect > 0.9; and 1, perfect [22]. The data analysis was carried out using the Statistical Package for the Social Sciences (SPSS for Windows, version 25, IBM Corp., Armonk, New York, NY, USA). Statistical significance was set at *p* < 0.05.

## 3. Results

**Figure 1:**
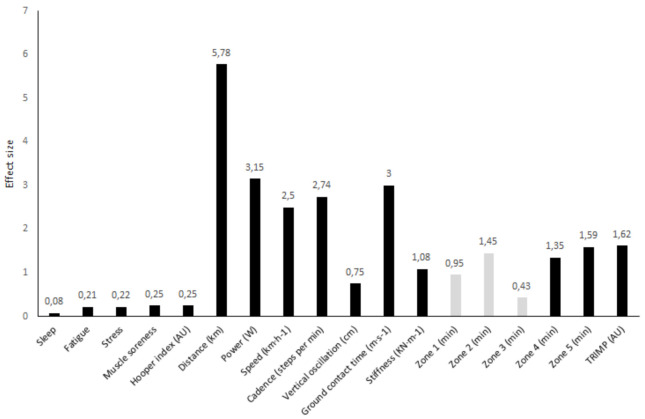
Effect sizes for the field referees’ (FR) and assistant referees’ (AR) differences in Hooper indices, external and internal match loads. Note. TRIMP = training impulse; black = higher values for FR; white = higher values for AR.

The differences in Hooper indices, external loads and internal loads recorded by FR and AR during official matches are shown in Table 1 and Figure 1. While no significant differences were found in Hooper indices between match officials, FR registered higher external loads (*p* < 0.01; ES: 0.75 to 5.78) in comparison with AR. In addition, FR spent more time in zone 4, zone 5 and recorded greater TRIMP values (*p* < 0.01; ES: 1.35 to 1.62) than AR. On the contrary, FR spent less time in zone 1, zone 2 and zone 3 (*p* < 0.01; ES: –0.43 to –1.45) compared with AR.

For FR, the initial fatigue and sleep subsets were positively correlated with power (*p* < 0.05; r = 0.44 to 0.52; CL = 0.27 to 0.29) and negatively with ground contact time (*p* < 0.05; r = −0.44 to −0.51; CL = 0.27 to 0.29). A moderate association between the initial stress subset and vertical oscillation was reported in (*p* < 0.05; r = 0.36; CL = 0.22) in ARs.

In FR, the sleep subset correlated positively with time spent in zone 5 and TRIMP value (*p* < 0.05; r = 0.43 to 0.46; CL = 0.29) and negatively with the time spent in zone 2 (*p* < 0.05; r = −0.48; CL = 0.28). In addition, the stress subset was associated negatively with time spent in zone 1 (*p* < 0.05; r = −0.42; CL = 0.30). The sleep subset correlated negatively with the time spent in zone 5 (*p* < 0.05; r = −0.33; CL = 0.22) and positively with the time spent in zone 1 (*p* < 0.05; r = 0.34; CL = 0.22) in ARs.

The associations between internal and external match loads for FR and AR are reported in Table 2. For FR, power and speed were negatively correlated with time spent in zone 2 (*p* < 0.05; r = −0.43; CL = 0.29). A positive association between ground contact time and zone 2 and zone 3 (*p* < 0.05; r = 0.50 to 0.60; CL = 0.24 to 0.27) was found. Moreover, power, speed, cadence and ground contact time correlated with time spent in zone 5 and TRIMP (*p* < 0.05 to 0.01; r = 0.42 to 0.64; CL = 0.22 to 0.30). A negative correlation between speed and the time spent in zone 1 (*p* < 0.05; r = −0.30; CL = 0.22) was found in AR.

## 4. Discussion

This is the first study that examined the associations between well-being status variables and relevant metrics representing match internal and external load in FR and AR. Indeed, previous studies only analyzed the differences in both external and internal load between FR and AR during official matches [19,23,24]. Furthermore, no investigations examined whether the perception of the initial well-being state can influence officials’ match external or internal loads.

Soccer match officials need to face competition at optimal physical conditioning to ensure that officials can cope with the high physical match demands by keeping up with play at all times to attain optimal positioning when making key decisions [25,26]. To our knowledge, this is the first investigation using the Hooper well-being tool in match officials before the official matches aiming to assess the initial players’ wellness state. Our results showed that there are no differences in well-being subsets between FR and AR before officiating matches, so it seems that both officials face the matches in similar conditions attending to the well-being state. However, the Hooper indices declared by FR and AR were moderate (≈2–3 points of 7 point scale). One of the main challenges of physical trainers of match officials is to prepare them to exhibit their optimal physical fitness during competition. The knowledge of the officials’ well-being state could help them to modulate the weekly load within the training sessions. In the literature we found that soccer players declared moderate values on every Hooper variable (between 2.5 and 3.10 points of a 7 point scale) [15], while other studies declared a larger range but with low–moderate values also (between 2.2 and 3.6 points of a 7 point scale) [13]. These results are in accordance with the values declared by the soccer match officials in our study—between 2.7 and 2.9 points of a 7 point scale in FR and between 2.4 and 2.8 points of a 7 point scale in AR. It could be interesting to implement strategies in FR and ARs’ weekly loads in order to help them to start matches more rested, less fatigued, without soreness and less stressed.

Regarding the external loads, this is the first investigation using Stryd devices on match officials. The external load results obtained by both FR and AR in the present study are in general lower than those obtained in previous studies. [2,3]. The main reason for the lower total distance covered by the FR and AR of our study in comparison to the literature [2,3], could be explained by the different competitive-level, because other studies analyzed the external loads in professional and national competitions, while the match officials involved in this study officiated at provincial level (i.e., *División de Honor*). FR covered higher distances and recorded greater power, mean speed, mean cadence, mean vertical oscillation, ground contact time and stiffness in comparison to AR. Therefore, considering Stryd has also detected the differences between FR and AR, it seems that Stryd is also a suitable device, like the GPS and multicamera video tracking, to quantify the external loads in match officials [3,27]. In addition, our results are also in consonance with those which reported higher internal loads (e.g., HR in periods of 15 min, HR_peak_, %HR_mean_) in FR in comparison to AR [2,9,19,23]. Specifically, in our study FR spent more time in zone 4 and zone 5 and recorded greater TRIMP value than AR. These findings suggest the usefulness of Stryd Power Meter as an alternative to GPS device aiming to monitor the external match loads because both pieces of equipment are able to detect the differences between FR and AR during official matches, which is likely because the activity of each AR is limited to one half of the field.

Analyzing the association between the initial well-being state and match loads could be relevant for the physical training of officials in order to know if the fact of facing matches more rested and less fatigued allows them to exhibit greater performance during match play [14,28,29]. Generally, in our study no correlations between most of the Hooper indices (stress, muscle soreness and Hooper index) and external loads variables were found for either FR or AR. However, power and ground contact time correlated with sleep and fatigue in FR, and the stress subset was associated with vertical oscillation in AR. Additionally, no associations between the Hooper indices and internal loads were found for officials. However, the sleep subset correlated with the time spent in zone 2 and 5, and TRIMP for FR, and the stress subset was associated with the time spent in zone 1. Thus, low and moderate influence between initial state and external and internal loads was found for FR and AR, respectively. However, considering that the magnitude of these associations is not large, it seems that FR and AR carry out regular training, controlled by a strength and physical specialist, allowing them to face official matches in optimal physical condition. Future research is needed to investigate whether fatigue, understood as bad sleep and high levels of stress, could affect the external or internal loads during official competition. This knowledge would be important for physical trainers in order to implement specific training sessions during the week, modulating the training loads in order to optimize the on-field officials’ performance and, consequently, reducing the injury risk.

Regarding the associations between the external and internal loads, in our study FR showed that power and speed were negatively correlated with time spent in zone 2, and ground contact time was positively correlated with time spent in zone 2 and 3. Moreover, distance, power, speed and ground contact time were correlated with zone 5 and TRIMP. These results coincide with those studies that report associations between the total distance and the TRIMP in amateur referees who officiated in Third Spanish Division [9]. Likewise, the magnitude of our correlations between internal and external loads were similar (e. g., small to moderate) to previous investigations in which GPS equipment was used [6,10]. As such, Catteral et al. [6] reported a low correlation (r = 0.15) between the total distance covered and HR_mean_ in English professional soccer matches, and Costa et al. [10] found moderate association (r = 0.22) between the total distance covered and TRIMP in international matches. In our study, moderate correlation (r = 0.32) has been found between the total distance covered and TRIMP during amateur matches. Therefore, the Stryd Power Meter can provide similar associations between external and internal loads to other equipment such as GPS or multicamera video tracking. In addition, the low and moderate associations reported could confirm the practical applications of other studies, which concluded that it is necessary to measure both external and internal loads because different dimensions are monitored. Additionally, there were no associations between the internal loads with vertical oscillation and stiffness, so it can be interesting to measure these variables since they could measure another dimension of the load and provide interesting data to program the training sessions of the FR. Regarding the association for AR, speed was negatively correlated with the time spent in zone 1, showing that the higher speed the lower the amount of time spent time in zone 1. To our knowledge, there is no evidence of a strong correlation between the Stryd variables and the internal load in soccer refereeing, so bearing in mind this is the first investigation which has proposed the use of the Stryd Power Meter to quantify external loads in match officials, it would be interesting to see whether more research could deepen in this topic. 

The present study is not exempt from limitations. Our experimental design limited us to only examining one competitive level, so our findings should be taken with caution. In this sense, it would be interesting to replicate this investigation at a higher level of standard of play in order to know if the Stryd Power Meter is also suitable to quantify external loads. Another limitation was the use of only objective method (e.g., heart rate) to quantify internal loads; however, other subjective tools, such as the rating of perceived exertion, have also been demonstrated to be valid and reliable to assess the internal load in match officials [30]. Otherwise, new variables were used in this study to quantify the well-being state and general fatigue before the official competition. Finally, it is important to use well-being variables (i.e., Hooper scale) and stiffness variables not only during official matches but also during training sessions in order to assess the daily load of match officials. Therefore, future research could analyze the association between training loads during the microcycle and the well-being state on the match day. 

## 5. Conclusions

The results of this study show that despite the fact that FR and AR declare similar values of Hooper variables, FR are exposed to a larger external and internal load than AR, probably because AR’s action space is limited. These results could suggest a necessity for the planning specific training programs for FR and AR to improve their physical performance. Furthermore, the initial well-being status seems not to be related with the performance because the correlations found were few in number and small or moderate both in FR and in AR. Finally, both in FR and AR, the external load and internal load are related, showing greater external loads when match officials spent more time in zone 5 and when they show greater TRIMP values.

## Figures and Tables

**Table 1:** Differences in Hooper indices, external and internal match loads between field referees (FR) and assistant referees (AR).

	FR (*n* = 23)		AR (*n* = 46)		Mean Differences (%)
	Mean ± SD	CV (%)	Mean ± SD	CV (%)	
Hooper indices					
Sleep	2.87 ± 1.17	40.92	2.78 ± 1.11	40.03	3.13
Fatigue	2.9 ± 1.07	36.7	2.67 ± 1.23	46.01	8.94
Stress	2.78 ± 1.55	55.78	2.48 ± 1.28	51.56	12.28
Muscle soreness	2.70 ± 1.27	47.11	2.41 ± 1.13	46.70	11.71
Hooper index (AU)	11.26 ± 3.98	35.38	10.35 ± 3.43	33.12	8.82
External loads					
Distance (km)	8.65 ± 0.84	9.72	3.92 ± 0.80	20.35	120.86 **
Power (W)	120.72 ± 11.75	9.74	89.70 ± 7.96	8.87	34.58 **
Speed (km·h^−1^)	7.11 ± 0.63	8.92	5.79 ± 0.43	7. 37	22.88 **
Cadence (steps per min)	62.83 ± 1.73	2.75	55.93 ± 3.30	5.90	12.33 **
Vertical oscillation (cm)	8.00 ± 0.54	6.69	7.58 ± 0.56	7.41	5.46 **
Ground contact time (m·s^−1^)	541.21 ± 57.73	10.67	346.02 ± 72.56	20.97	56.41 **
Stiffness (KN·m^−1^)	9.28 ± 0.56	6.06	8.66 ± 0.59	6.78	7.16 **
Internal loads					
Zone 1 (min)	1.36 ± 3.35	245.16	15.05 ± 25.61	170.18	−90.93 **
Zone 2 (min)	4.51 ± 10.20	226.31	23.07 ± 15.48	67.08	−80.47 **
Zone 3 (min)	21.73 ± 16.19	74.51	27.65 ± 11.60	41.97	−21.40 **
Zone 4 (min)	41.69 ± 12.05	28.90	22.51 ± 16.30	72.39	85.20 **
Zone 5 (min)	24.36 ± 13.88	57.00	7.37 ± 7.44	101.01	230.54 **
TRIMP (AU)	364.11 ± 44.65	12.26	267.47 ± 74.75	27.95	36.13 **

Note. SD: standard deviation; CV: interplayer coefficient of variation; TRIMP = training impulse; ** Significance level set at *p* < 0.01.

**Table 2:** Relationships (r/rho; ± 90% CL) between external and internal match loads for field (FR) and assistant referees (AR).

Variables	Officials	Distance	Power	Speed	Cadence	Vertical Oscillation	Ground Contact Time	Stiffness
Zone 1	FR	−0.09; ± 0.35?	−0.14; ± 0.35?	−0.21; ± 0.34S	−0.17; ± 0.34S	−0.14; ± 0.35?	0.22; ± 0.34S	−0.17; ± 0.34S
	AR	−0.15; ± 0.24S	−0.23; ± 0.23S	−0.30; ± 0.22S *	−0.08; ± 0.24?	0.11; ± 0.24S	0.05; ± 0.25?	0.16; ± 0.24S
Zone 2	FR	−0.21; ± 0.34S	−0.43; ± 0.29M *	−0.42; ± 0.30M *	−0.37; ± 0.31 M	−0.10; ± 0.35?	0.60; ± 0.24L **	0.10; ± 0.35?
	AR	−0.26; ± 0.23S	−0.06; ± 0.24?	−0.13; ± 0.24S	−0.07; ± 0.24?	0.06; ± 0.24?	−0.16; ± 0.24S	0.00; ± 0.25?
Zone 3	FR	−0.23; ± 0.34S	−0.39; ± 0.30M	−0.35; ± 0.31M	−0.25; ± 0.33S	−0.24; ± 0.33S	0.50; ± 0.27M *	0.24; ±0.33S
	AR	−0.11; ± 0.24S	0.12; ± 0.24S	0.22; ± 0.23S	−0.14; ± 0.24S	0.24; ± 0.23S	−0.11; ± 0.24S	−0.21; ± 0.24S
Zone 4	FR	−0.13; ± 0.35?	0.07; ± 0.35?	0.07; ± 0.35?	−0.05; ± 0.35?	0.23; ± 0.34S	−0.33; ± 0.32M	−0.02; ± 0.35?
	AR	0.18; ± 0.24S	0.12; ± 0.24S	0.22; ± 0.23S	0.09; ± 0.24?	−0.11; ± 0.24S	−0.02; ± 0.25?	0.02; ±0.25?
Zone 5	FR	0.48; ± 0.28M *	0.64; ± 0.22L **	0.58; ± 0.24L **	0.42; ± 0.30M *	0.18; ± 0.30S	−0.52; ± 0.27L *	−0.02; ± 0.35?
	AR	0.19; ± 0.24S	0.12; ± 0.24S	0.17; ± 0.24S	0.10; ± 0.24?	0.02; ± 0.25?	0.04; ± 0.25?	0.05; ± 0.25?
TRIMP	FR	0.32; ± 0.32M	0.55; ± 0.26L **	0.48; ± 0.28M *	0.38; ± 0.31M	0.34; ± 0.32M	−0.59; ± 0.24L **	0.02; ± 0.35?
	AR	0.18; ± 0.24S	0.21; ± 0.24S	0.29; ± 0.23S	0.06; ± 0.24?	−0.01; ± 0.25?	−0.02; ± 0.25?	−0.10; ± 0.24S

Note. CL = Confidence limits; TRIMP = training impulse; * Significance level set at *p* < 0.05; ** Significance level set at *p* < 0.01; Correlation magnitude; ?: unclear; S: small; M: moderate; L: large; VL: very large; NP: nearly perfect.
